# Perturbations in the carbon budget of the tropics

**DOI:** 10.1111/gcb.12600

**Published:** 2014-06-06

**Authors:** John Grace, Edward Mitchard, Emanuel Gloor

**Affiliations:** 1 Schoool of GeoSciences, The University of EdinburghEdinburgh, EH9 3JN, UK; 2 The School of Geography, University of LeedsLeeds, LS2 9JT, UK

**Keywords:** carbon emissions, deforestation, forest degradation, human development index, land-use change

## Abstract

The carbon budget of the tropics has been perturbed as a result of human influences. Here, we attempt to construct a ‘bottom-up’ analysis of the biological components of the budget as they are affected by human activities. There are major uncertainties in the extent and carbon content of different vegetation types, the rates of land-use change and forest degradation, but recent developments in satellite remote sensing have gone far towards reducing these uncertainties. Stocks of carbon as biomass in tropical forests and woodlands add up to 271 ± 16 Pg with an even greater quantity of carbon as soil organic matter. Carbon loss from deforestation, degradation, harvesting and peat fires is estimated as 2.01 ± 1.1 Pg annum^−1^; while carbon gain from forest and woodland growth is 1.85 ± 0.09 Pg annum^−1^. We conclude that tropical lands are on average a small carbon source to the atmosphere, a result that is consistent with the ‘top-down’ result from measurements in the atmosphere. If they were to be conserved, they would be a substantial carbon sink. Release of carbon as carbon dioxide from fossil fuel burning in the tropics is 0.74 Pg annum^−1^ or 0.57 MgC person^−1^ annum^−1^, much lower than the corresponding figures from developed regions of the world.

## Introduction

The tropical land surface has undergone substantial changes in the last few decades as forest has been cleared to enable other forms of land use. These changes involve the energy balance, the cycling of carbon and water and emissions of greenhouse gases at local and regional scales; they are believed to be sufficiently large to influence the climate system. Simulations using climate models suggest these changes will lead to significant feedbacks to the climate system, including an increase in temperature and a decrease in regional precipitation (Lean & Warringlow, 1989; Shukla *et al.*, 1990; Costa & Foley, 2000; Voldoire & Royer, 2004; Medvigy *et al.*, 2011; Ehn *et al.*, 2014). Precipitation over tropical land masses could decrease significantly and possibly weaken the tropical atmospheric circulation (Vecchi *et al.*, 2006), and these climatic effects may teleconnect to other parts of the world (Werth & Avissar, 2002). Consequently, interest in the carbon balance of the tropics, and especially the impact of deforestation on the carbon cycle, remains high (Ciais *et al.*, 2010; Gloor *et al.*, 2012; Patra *et al.*, 2013; Aragão *et al*., 2014).

Most of the land surface change in the tropics is driven by the need to clear forests and woodlands to provide agricultural land (Montoya & Rull, 2011; Galan de Mera *et al.*, 2012; Houghton *et al.*, 2012) and also to satisfy a growing demand for timber, fibre and – more recently – biofuel (Koh & Wilcove, 2008; Aragão *et al*., 2014). Public attention is usually focussed on the loss and degradation of pristine tropical forests, which are often presented in media reports as ‘the lungs of the Earth’ as they exchange huge volumes of gases with the atmosphere (Laurance, 1999). However, the tropics contain other ecosystems too, most notably secondary forests, savannas (woodlands and grasslands), mangroves, plantations and many forms of agriculture. Although these normally contain less carbon per area than intact rain forests, they nevertheless must be considered in any attempt to make a comprehensive analysis of the carbon fluxes and especially the effect of the pressure placed on the tropics by an increasing population of human consumers.

Our overall knowledge of carbon stocks and fluxes has increased hugely in recent years, as a result of new tools for research, and a vigorous and multi-disciplinary approach to the detection of land-use change. Most noteworthy have been (i) the attempts to measure carbon stock changes of intact forests using networks of sample plots (Phillips *et al.*, 1998; Malhi *et al.*, 2002; Lewis *et al.*, 2009), (ii) the introduction of micrometeorological methods to estimate net carbon fluxes at representative tropical forests (Grace *et al.*, 1995; Kruijt *et al.*, 2004; Saleska *et al.*, 2009), (iii) innovation in satellite remote sensing for the detection of change in land cover and mapping of carbon stocks (Achard *et al.*, 2002; Asner *et al.*, 2010; Saatchi *et al.*, 2011; Baccini *et al.*, 2012; Hansen *et al.*, 2013; Mayaux *et al.*, 2013), (iv) measurements of carbon dioxide concentration in the atmosphere to infer carbon fluxes at a very large scale (Jacobson *et al.*, 2007; Gatti *et al.*, 2010, 2014; Sarmiento *et al.*, 2010) and (v) the use of field experimentation to assess the effect of drought and disturbance on carbon stocks, fluxes and ecosystem vulnerability (San José *et al*., 2003; Nepstad *et al.*, 2007; Lloyd *et al*., 2009; Da Costa *et al.*, 2010; Meir & Woodward, 2010; Don *et al.*, 2011; Salinas *et al.*, 2011). Despite these efforts, major uncertainties in the overall carbon budget of the tropics remain (House *et al.*, 2003; Ziegler *et al.*, 2012; Wright, 2013), and there are strong regional variations which depend on government policies and changing patterns of global demand for products (Ciais *et al.*, 2007, 2010; Williams *et al.*, 2007; Achten & Verchot, 2011; Gloor *et al.*, 2012). In this article we examine progress made in the last 10 years to define the stocks and fluxes of carbon in the tropics, and to understand the natural and anthropogenic drivers of change.

## The terrestrial surface

The terrestrial surface of the tropics is defined as the land between latitudes 23.44°N and 23.44°S; it covers some 44 million km^2^, scattered between 93 major countries in tropical parts of Africa, Asia and South and Central America. It constitutes 8.6 per cent of the planetary surface and 30% of the global land surface; it varies in elevation from sea level to 6768 m in the Peruvian Andes.

The land on all continents except Antarctica is to some extent vegetated and thus takes up substantial amounts of carbon dioxide (CO_2_) from the atmosphere through the process of photosynthesis, most of which is ultimately released from ecosystems back to the atmosphere via respiration. Globally, the mass of carbon absorbed by photosynthesis, the Gross Primary Productivity, GPP, is huge, slightly more than 120 Pg C annum^−1^ (Lieth & Box, 1977; Roy *et al.*, 2001; Beer *et al.*, 2010). Most of this uptake occurs in the tropics, where the GPP is estimated to be as high as 72 Pg C annum^−1^ (Beer *et al.*, 2010).

Ecosystems store carbon as macromolecules (lignin, cellulose, starch, proteins) in plants and soil for a variable period of time (Galbraith *et al.*, 2013), subsequently releasing CO_2_ through plant, animal and microbial respiration, and fire. The combined efflux to the atmosphere is somewhat less than the uptake by gross photosynthesis. We know this from changes in the concentrations of gases and their isotopes in the atmosphere, and from models (Falkowski *et al.*, 2000; Roy *et al.*, 2001). In former times these two opposing fluxes may sometimes have been in equilibrium i.e. the uptake of CO_2_ by photosynthesis averaged over a number of years might well have been balanced by the loss of CO_2_ from respiration and fire.

Nowadays the carbon cycle is out of equilibrium as a result two classes of major perturbations. The first, perpetrated by the rich countries of the world, is the inexorable increase in fossil fuel burning; the second is the removal of forests in tropical countries. According to most authorities, the carbon released by deforestation rose sharply in the 1980s but has decreased somewhat in recent years (Houghton *et al.*, 2012). In 2010, it was thought to be less than 1 PgC annum^−1^ while the global fossil fuel emissions were still on the increase, recently standing at 8.7 PgC annum^−1^ (Boden *et al.*, 2010; Friedlingstein *et al.*, 2010). Geological processes such as weathering and volcanism also contribute to the exchange of CO_2_ between the land and atmosphere but their contribution is only important on a geological time scale (Berner, 2003).

Apart from CO_2_, other carbon species may be significant. In particular, methane emissions have changed in recent decades as a result of human activity. A full discussion of methane is outside the scope of this article, and can be found elsewhere (Kirschke *et al.*, 2013). The global emission of methane is between 0.55 and 0.68 Pg CH_4_ annum^−1^ and according to recent satellite observations the tropical component is 0.20 Pg CH_4_ annum^−1^ (Frankenberg *et al.*, 2008). Most of the tropical component is probably from natural aquatic ecosystems, but some is associated with land-use change, particularly the increase in rice production, biomass burning and the flooding of the land to create reservoirs (Reay *et al.*, 2010; Fearnside & Pueyo, 2012).

A century ago, when human influences were less than today, we may guess that all 44 million km^2^ of the tropics, with the exception of high mountains and a few dry areas, would have supported some kind of tree cover. Now, less than 18 million km^2^ of forest remain (FAO, 2011) and deforestation continues albeit at a reduced rate compared to the period 1960–1980, while agricultural land keeps expanding (Houghton *et al.*, 2012). But in experiments where tropical grasslands and savannas are protected from fire and grazing, trees return and the land usually becomes forested within a few years (Bond *et al.*, 2005; Grace *et al.*, 2006). Another way in which humans are thought to be influencing tropical forests and woodlands is by the increase in concentrations of CO_2_ derived from fossil fuel burning. This increase may be stimulating photosynthesis, which may contribute significantly to uptake of atmospheric CO_2_ (Lloyd & Farquhar, 1996; Lloyd, 2002; Pan *et al.*, 2011) and may explain the observation that biomass in undisturbed forest plots is increasing (Phillips *et al.*, 1998; Lewis *et al.*, 2009). The topic is however controversial as large scale experimentation on the effects of CO_2_ enrichment on tropical forests has not yet been achieved (but see Tollefson, 2013) and estimates of the CO_2_ effect rests on work carried out in microcosms (although some microcosms have been rather large, Rosenthal, 1998; Krner & Arnone, 1992), or theoretical considerations from physiological and biochemical knowledge (Lloyd & Farquhar, 1996). Sometimes, increases in biomass in sample plots (Phillips *et al.*, 1998; Lewis *et al.*, 2009) are considered to be evidence of the impact of rising CO_2_, but clearly other factors may also be causing such changes (Wright, 2013).

Forests and woodlands contain large and conspicuous stocks of above-ground carbon, and when they are cleared to make way for other land uses, much of this carbon is lost to the atmosphere as CO_2_. The land area that can be deemed ‘forest’ clearly depends on the operational definition of ‘forest’. The Food and Agriculture Organization (FAO) defines forest as ‘land with a tree cover of more than 10 per cent and an area of more than 0.5 ha’. The definition further states: ‘the trees should be able to reach a height of 5 m at maturity *in situ*’ (FAO, 2000). It excludes land that is ‘predominantly used for agriculture’, but clearly includes most woodland savanna but not grassland savanna.

The published areas of tropical lands covered with forests, pastures and crops may be obtained from FAO statistics and satellite remote sensing (Table 1), although the various definitions of ‘tropical forest’ adopted by authors lead inevitably to confusion and contradictory figures (see Lambin *et al*.,2003; Gibbs *et al.*, 2007; Hansen *et al.*, 2013). Here, we decided to use satellite data from the European Space Agency (ESA) as it is readily available and uses the United Nations Land Cover Classification System.

**Table 1:** Wide variation in the reported area of tropical forests (including open forests and dry forests). FAO (2011) uses country reports, the others are from the interpretation of remote sensing data. Lewis *et al*. (2009) used the average of four different data sets including FAO and remote sensing, Globcover2009 refers to a global map produced by the European Space Agency using satellite data from January to December 2009, Saatchi *et al*. (2011) used various remotely sensed data products. Units are millions of km^2^

	FAO (2011)	Lewis *et al*. (2009)	GlobCover2009	Saatchi *et al*. (2011)	Mean
America	8.90	7.87	9.90	12.1	9.69
Africa	5.95	6.32	9.78	7.75	7.45
Asia	2.94	3.58	1.98	4.74	3.31
Total	17.79	17.77	21.66	24.59	20.45

The ESA GLOBCOVER project created land-cover maps using observations from the MERIS sensor (300 m resolution) on board the ENVISAT satellite mission for periods between December 2004 and December 2009. It appears from the 2009 product that tropical lands are about 47% forest, with 26% pasture and 10% croplands (Table 2). The remaining land falls outside these definitions, and includes other vegetated areas, floodplains and urban complexes, and also grasslands with scattered trees below the 10% canopy cover threshold. There has been a progressive loss of tropical forest over the last 50 years, related mostly to the extent of human development which has steadily increased in parallel with population growth (DeFries *et al.*, 2010).

**Table 2:** Land use for crops, pastures and forests in the tropics. Data for crops and pastures are from Ramankutty *et al.,* (2008) and are based on country data reported from years 1996 to 2003; data for forests are from the last column of Table 1. Note that the residual ‘Other’ for Asia is negative, implying some degree of confusion or overlap in the reported classification between forest and non-forest

Region	Total land area (million km^2^)	Cropland (million km^2^)	Pasture (million km^2^)	Forest (million km^2^)	Other
America	16.36	1.24	3.98	9.69	1.52
Africa	22.97	1.94	7.28	7.45	6.30
Asia	3.80	0.97	0.06	3.31	−0.54
Total	43.13	4.15	11.32	20.45	7.21

Other natural and semi-natural ecosystems of the tropics include grasslands with various fractions of tree cover and contain substantially less carbon per area than forests, although sometimes have a larger stock of carbon below-ground as soil organic matter (Juo *et al.*, 1995; San José & Montes, 2001; Fisher *et al.*, 2007; Saiz *et al.*, 2012). Mangroves for example are reported to have extremely high carbon stocks per area, averaging 1023 Mg C ha^−1^ when the below-ground component is included, with some of the highest values of Net Primary Productivity ever recorded (Donato *et al.*, 2011), but their global area is only 200 thousand km^2^, just 0.5% of the land in the humid tropics. Forming a fringe between the lands from the ocean, they export significant quantities of recalcitrant carbon compounds to the sea (Silva *et al.*, 1991; Kristensen *et al.*, 2008; Tamooh *et al.*, 2008; Alongi, 2012). This, along with the export of plant products, is an example of lateral transport of carbon from the tropics (see Gloor *et al.*, 2012).

**Figure 1:**
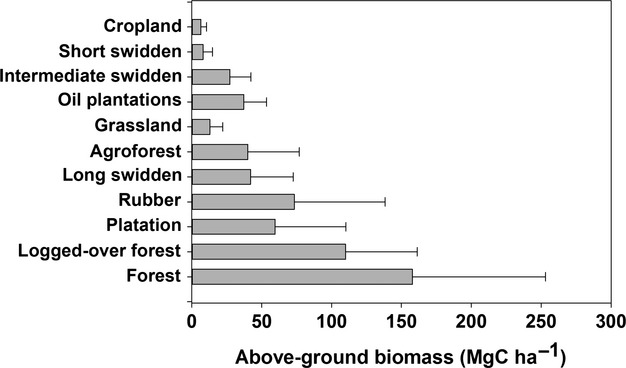
Statistical spread of above-ground Biomass for 11 types of land use in the tropics, plotted from the data of Ziegler *et al*. (2012). Box plots represent medians and quartiles, SD and outliers.

Ziegler *et al*. (2012) reported the range of above-ground carbon per area of tropical ecosystems to vary from a few tonnes per hectare to over 400 Mg C ha^−1^ (Fig.1). The data show that forests contain more above-ground carbon than the various cropped systems and suggest that deforestation will inevitably result in a large loss of carbon.

Substantial stocks of carbon occur in tropical soils as Soil Organic Carbon (SOC). Estimates by soil scientists for the carbon stored as SOC in the entire tropics vary from less than 200 Pg (Amundson, 2001; FAO, 2010) to 500–650 Pg (Eswaran *et al.*, 1993; Sombroek *et al.*, 1993; Batjes, 1996). One reason for the large discrepancies in the literature is that some authors consider the soil carbon only in the surface layers rather than in the whole soil profile, assuming the organic matter below the arbitrary depth of 1 m to be inactive. Carbonate-carbon is also significant in some regions (Batjes, 1996), and so is charcoal remaining from long-past slash and burn; however this latter component is considered by most authorities to be inert (but see Bird *et al.*, 1999). If we take Batjes's figures of 616–640 Pg for the SOC in the top 2 m, and divide by the area of tropical lands (taken as 48 million km^2^), we may conclude that tropical lands have, on average 128–133 MgC ha^−1^ of SOC. Alternatively if we consider only the top metre of soil we have 80–84 MgC ha^−1^, a figure which is close to the average found by researchers in the field (Don *et al.*, 2011).

What happens to this soil organic carbon when land use is changed is not always clear (Table 3, data from Don *et al.*, 2011). Eclesia *et al*. (2012) showed that when forests are replaced by plantations of pine or eucalyptus, the soil carbon content increases linearly, and after a century it far exceeds the levels found in the native forest, except in the wettest sites. It is often proposed that plantations may be used to rehabilitate degraded lands, as they may increase the carbon and nutrient content in the surface layers (Chazdon, 2008); moreover, long-lived and slow-growing plantation species are found to have soils with remarkably high carbon content (Kraenzel *et al.*, 2003). However, in some cases, conversion of native forest to fast-growing commercial plantations such as cocoa, coconut and oil palm is reported to cause a decline in soil carbon (Chiti *et al.*, 2014). The whole issue of the carbon balance of tropical plantation requires further research, to be co-ordinated across regions and to be funded by agencies which have no vested interest in the results.

**Table 3:** Carbon storage in soil organic carbon (SOC) and the change resulting from a transition in land use (from Don *et al.*, 2011). Negative denotes loss of organic matter, positive denotes gain. ± shows the SE of the Mean and *n* is the number of observations. The data are based on paired sample plots, the time after the transition varies from 21 and 49 years

Transition	SOC before change (Mg ha^−1^)	Change in SOC (Mg ha^−1^)	*n*
Primary forest to grassland	73 ± 7	−12.6 ± 3.0	93
Primary forest to cropland	83 ± 9	−20.1 ± 5.2	56
Primary forest to perennial crops	105 ± 20	−32.0 ± 3.5	20
Primary forest to secondary forest	91 ± 9	−12.6 ± 2.4	71
Secondary forest to grassland	84 ± 6	−11.0 ± 3.4	66
Secondary forest to cropland	88 ± 12	25.8 ± 6.9	26
Secondary forest to perennial crops	90 ± 17	5.6 ± 3.0	15
Grassland to secondary forest	60 ± 9	+ 12.4 ± 6.1	32
Cropland to secondary forest	70 ± 9	+ 33.2 ± 10.5	25
Grassland to cropland	64 ± 15	+ 6.0 ± 5.8	15
Cropland to grassland	61 ± 17	+ 7.6 ± 5.8	16
Cropland to fallow	43 ± 7	+ 8.9 ± 2.9	21

Contrary to common expectations, the forest-to-pasture transition may sometimes lead to an increase in soil carbon, especially in the surface layers of soil (Guo & Gifford, 2002; Don *et al.*, 2011; Eclesia *et al.*, 2012; Yonekura *et al.*, 2012) as suspected from earlier research on South-American pastures made up of introduced grasses (Fisher *et al.*, 1994). Often, the largest increases are in the wetter sites. This analysis is intriguing and relevant to much of the land-use change occurring in the tropics today, but the difference in behaviour between the various types of transition have not been explained, and results may be quite different for other plantation types (coconut, oil palm, cacao). Marin-Spoitta & Sharma (2013) found trends in soil carbon that were weaker, with an overwhelming effect of mean annual temperature and precipitation. In an extensive review of soils of sub-Saharan Africa, Vågen *et al*. (2005) concluded that largest potential for increasing SOC is through the establishment of natural or improved fallow systems (agroforestry), which provide rates of C sequestration in the range of 0.1–5.3 MgC ha^−1^ annum^−1^.

### Forest biomass derived from national inventories

Prompted by the 1992 United Nations Conference on Environment and Development (UNCED), the Food and Agriculture Organization (FAO) publishes a biennial series called *State of the World's Forests* in which data on forest areas reported by national governments are compiled. Recommendations for standardized reporting of carbon stocks of forests based on ecosystem mensuration are described in several publications including Penman *et al*. (2003) and GOFC-GOLD (2010). The FAO data on forest area (Table 4) are widely cited by researchers as a source of information on deforestation, and the 2011 report contains additionally a table of biomass carbon changes derived from such data (FAO, 2011). Taken at face value, these figures show an annual carbon flux from deforestation in the period from 2000 to 2010 of 0.88 PgC annum^−1^. However, reporting has not been consistent and the quality of national data is often questioned (Achard *et al.*, 2002; Grainger, 2008; Gloor *et al.,* 2012). National inventory data sets are being rapidly superseded by satellite data, as discussed in a later section.

**Table 4:** Areas and carbon stocks of tropical forests from 1990 to 2010 according to FAO (2011). Also included are the carbon stocks in dead wood and litter (‘litter’) and soil according to FAO

	Areas (millions of km^2^)				Carbon stocks in biomass (PgC)				Litter (PgC)	Soil (PgC)
	1990	2000	2005	2010	1990	2000	2005	2010	2010	2010
America	9.78	9.32	9.09	8.90	110.9	106.2	103.9	102.1	10.0	75.5
Africa	6.64	6.29	6.12	5.95	60.9	58.3	57.1	55.9	7.9	34.5
Asia	3.25	3.01	2.99	2.94	29.1	27.5	26.5	25.2	1.0	16.5
Total	19.76	18.63	18.20	17.81	200.2	192.0	187.5	183.2	18.9	126.5

### Forest biomass derived from research plots

Meanwhile, independent researchers have been pooling data from their own permanent sample plots, which are typically 1 ha marked areas in which all trees greater than 10 cm in diameter have been tagged and repeatedly measured using a common protocol (Phillips *et al.*, 1998; Malhi *et al.*, 2002; Lewis *et al.*, 2009). Such data are now vital for the calibration of remotely sensed information. Perceived limitations of the use of these 1 ha plots are (i) samples may not be entirely representative of the range of tropical forests, considering that many forests, even when they appear pristine, are actually in stages of recovery from disturbance, both natural and anthropogenic (Phillips *et al.,* 2002; Fisher *et al.*, 2007; Lloyd *et al.*, 2007; Gloor *et al.*, 2009) (ii) the allometric equations used to convert tree diameter and height to biomass introduce uncertainties especially for the large trees which usually contain most of the carbon (Chave *et al.*, 2005) (iii) further allometric relationships are required to estimate below-ground biomass, although below-ground biomass data are scarce because of the practical difficulties of achieving large and representative samples (Kenzo *et al.*, 2009; Ryan *et al.*, 2011) (iv) soil carbon stocks as soil organic matter and elemental carbon are not generally recorded, and if they are, the studies are confined to surface layers only. Despite these difficulties, analysis of some 156 sample plots covering an area of 163 ha on three continents suggests these forests have been accumulating carbon in recent decades at an average rate of 0.49 MgC ha^−1^ annum^−1^ (Lewis *et al.*, 2009).

### Forest biomass from satellite remote sensing

Satellite observations of land-use change in the tropics were first used on a large scale for the detection of deforestation in Brazil (INPE, 2003). These observations made use of the NASA Landsat satellites, which began with Landsat 1 in 1972 and continue today with Landsat 8. Other satellite missions widely used include the European SPOT (Système Pour l'Observation de la Terre) series of satellites, commencing with SPOT 1 (1986) and leading to SPOT 6 (launched 2012), which has acquired images with relatively high spatial resolution (10 m or less); ENVISAT which flew the Moderate Resolution Imaging Spectrometer MERIS from 2002 to 2012; the NOAA Advanced Very High Resolution Radiometer (AVHRR) from 1978 to the present; and NASA's Moderate resolution Imaging Spectroradiometer (MODIS) on board the Terra and Aqua satellites from 2000 to the present.

All of these instruments detect energy from the sun and sky that has been reflected from the planetary surface, in specific wavebands. Forest and ‘non-forest’ areas have different spectral signatures and so may be distinguished from each other in ideal conditions. But in the tropics the use of such optical remote sensing has been limited because of the frequent presence of clouds. Mayaux *et al*. (2013) describe how cloud-free data can nevertheless be attained by carefully selecting the images and using multiple satellites to obtain cloud-free images representing one year. Thus, satellites have provided valuable indications of the decline of forest cover over three decades (Achard *et al.*, 2002; Hansen *et al.*, 2013; Mayaux *et al.*, 2013).

Difficulties arise when we try to make comparisons between satellite-based estimates by different authors because quite different criteria to recognize ‘forest’ have been used (compare for example Achard *et al.*, 2002; Mayaux *et al.*, 1998 and Hansen *et al.*, 2010). This leads to totally different areas of forest, particularly in Africa where woodland savanna is a large part of a continuum that includes open forest and closed forest. If we define ‘forest’ broadly as having a canopy cover of 10% or more, as the FAO has done, the area of tropical forest from satellite data is 24.6 million km^2^ (Saatchi *et al.*, 2011), somewhat more than the 20 million km^2^ identified by the FAO methodology. But Achard *et al*. (2002) adopt a more restricted definition called ‘humid tropical forest’, which excludes dry forests and woodlands, and they find only 11.5 million km^2^.

Some of the difficulties of estimating forest areas and carbon stocks from space have been overcome by new technological developments. The first of these is the deployment, from space, of active radar remote sensing, which offers the possibility of not only detecting the extent of forest but of also estimating biomass from the back-scattered radar signal (Quegan *et al.*, 2000). Because radar sensors can ‘see’ the land surface even at night and when there is cloud cover, more data are acquired than with optical sensors. Moreover, radar penetrates the forest canopy to an extent which depends on its wavelength, and the back-scattered signal provides information on the amount of biomass per area of land (Le Toan *et al.*, 2011; Woodhouse *et al.*, 2012). From 2006 to 2011 the *Japanese Advanced Land Observing Satellite* (ALOS) carried a synthetic aperture radar sensor (PALSAR, the Phased Array type L-band Synthetic Aperture Radar) which has been used to map biomass distribution in Africa and elsewhere (Mitchard *et al.*, 2009; Ryan *et al.*, 2012; Reich *et al.*, 2013).

The second recent development is the use of space-borne LIDAR to measure the height of the vegetation and thus to estimate biomass from ground-based calibration data. The NASA satellite ICESat, designed primarily to measure the changing mass of polar ice sheets using LIDAR, flew from 2003 to 2009 and provided point estimates of tropical forest mass across the tropics, which were spatially extrapolated into the first pan-tropical maps of aboveground carbon using ancillary full-cover datasets (Saatchi *et al.*, 2011; Baccini *et al.*, 2012). Both studies used the ICESat data in combination with remotely sensed information on forest cover to model and map the spatial distribution of biomass across three continents for the 2000s.

However, these maps are not exactly the same, despite their common ICESat origin. The differences are likely to arise partly from the fact that the calibration data were from different field plots and the allometric equations used by the two groups working independently were not the same. Very recently, Mitchard *et al*. (2013a, b) have compared the two sets of results. On a per country basis or on a biomass density basis they agree to within 15 per cent, but when compared to the corresponding FAO data both sets of LIDAR data show significantly more biomass carbon than estimated from inventories. Overall, the total above-ground biomass in the tropics is 179 Pg of carbon according to the FAO inventory data, 203 Pg according to Saatchi *et al*. (2011), and 228 Pg according to Baccini *et al*. (2012). The apparent underestimation by the FAO data is noteworthy, and does significantly affect the estimates of global deforestation flux. We may estimate the below-ground biomass using an ‘expansion factor’ of 1.26 calculated from Luyssaert *et al*. (2007), and derive the total biomass carbon in the tropics as 256–287 Pg. Alternatively, if we take the FAO figures (Table 3) we find 202.1 Pg of carbon in biomass (with litter) and a further 126 Pg of carbon as soil organic matter. The very recent satellite data set from Hansen *et al*. (2013) does not agree well with the FAO figures, and is more consistent with Saatchi *et al*. (2011) and Baccini *et al*. (2012).

### The nature of the sources and sinks of carbon

**Figure 2:**
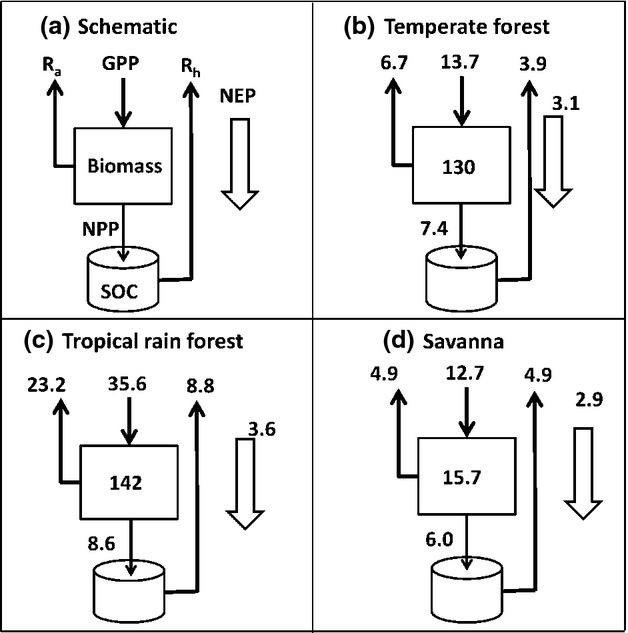
Typical carbon fluxes for temperate and tropical forests, and tropical savannah, showing Gross Primary Productivity (GPP), Net Primary Productivity (NPP), R_a_ (autotrophic respiration), Rh (heterotrophic respiration) and the overall carbon balance Net Ecosystem Production (NEP). Units are MgC ha^−1^ annum^−1^ for fluxes and MgC ha^−1^ for biomass stocks (shown in the central box). Based on data from Luyssaert *et al*. (2007).

Sources and sinks of atmospheric carbon arise when the input of carbon into the land surface does not equal the sum of all the carbon outputs. The main carbon input is by photosynthesis of green plants and the main outputs are autotrophic (i.e. plant) respiration R_a_, heterotrophic (i.e. microbe and animal) respiration R_h_ and fire. In the several hundred ecosystems of the world which have been investigated, the annual sum of photosynthesis, known as the Gross Primary Productivity GPP, usually exceeds R_a_ + R_h_ (Luyssaert *et al.*, 2007, 2008), implying that ecosystems are often carbon sinks at least at the local scale and over short (a few years) time scales. In a few investigations, ecosystems have been found to be a carbon source, especially following disturbance (Saleska *et al.*, 2003). Such studies provide useful insights into processes, but they are short term and thus generally do not pick up rare catastrophic events such as storms and fires, when forests suffer periodic reductions in biomass; these events occur particularly in relation to climate extremes associated with the Southern Oscillation (Aragão *et al*., 2008; Flores *et al.*, 2014). The data base of Luyssaert *et al*. (2007) provides consistent quality-controlled information on the constituent carbon fluxes for forest ecosystem under undisturbed conditions, enabling us to comment on the magnitudes of the tropical forest fluxes (Fig.2). We see that the photosynthetic input (GPP) of a typical tropical forest is as high as 32 MgC ha^−1^ annum^−1^, much higher than that of deciduous forest, presumably because conditions for photosynthesis are favourable all the year round in the humid tropics, whereas in other parts of the world there are climatological limitations for part of the year. However, respiratory effluxes R_a_ and R_h_ are also much higher in the tropical forest, so the Net Primary Productivity (GPP-R_a_) is usually between 5 and 10 MgC ha^−1^ annum^−1^ (see also Malhi, 2012) not particularly high compared to other ecosystems in both the tropics and the temperate zone (Clark *et al.*, 2001; Scurlock & Olson, 2002), and not nearly as high as tropical grassland sometimes can be (Long *et al.*, 1989). One reason why NPP of tropical forests is not much higher than for temperate forests is that almost all the temperate forests are in a relatively juvenile phase, being managed to be productive and containing trees which are in their most active growth phase (Luyssaert *et al.*, 2008).

Savanna ecosystems, with or without tree cover, contain much less biomass than rain forests and have a large fraction of biomass underground, a characteristic which enables them to recover from fire (Grace *et al.*, 2006; Ribeiro *et al.*, 2011). They have smaller annual fluxes, because growth is constrained by a long dry season in which many species shed their leaves (Sankaran *et al.*, 2005). They are also frequently burned and so suffer periodic reductions in leaf area. In the wet season, they may have a rather high rate of carbon assimilation, partly as a result of the ground cover of C_4_ grasses (Miranda *et al.*, 1997; San José *et al*., 2003; Santos *et al.*, 2003, 2004; Veenendaal *et al.*, 2004), which appear to contribute as much as 59% of the Primary Productivity of savannas world-wide (Lloyd *et al.*, 2008).

### Can the anthropogenic sources and sinks be deduced from existing data?

The ICESat-derived maps may have indeed provided the most reliable estimates of forest biomass; however, to track changes in the carbon stocks over decades one requires a long-term data set. So far, only the FAO data provide such a time series covering the whole tropics, although there are well-documented satellite data over long periods for particular areas, for example South America (Gloor *et al.*, 2012). From the available data, we may estimate the total flux arising directly from human perturbations as the sum of the constituent terms, each one representing a type of disturbance: 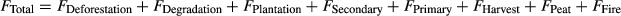 In the following paragraphs, we consider the terms one-by-one.

#### *F*_Deforestation_ the deforestation flux

To estimate the deforestation flux requires data on forest area of the entire tropics from more than one point in time. We have extracted the time series from FAO (2011). We present three estimates of deforestation flux (Table 5). The first, from FAO (2011) shows evidence of a decline in deforestation flux from 2000 to 2010, and stands at 0.80 Pg C annum^−1^ for 2005–2010. The second is a modification of the FAO data, obtained as the product of the area of forest ‘lost’ and the carbon content per area of land (MgC ha^−1^), obtained from regional average data calculated from Saatchi *et al*. (2011). The deforestation flux for the tropics for the period 1990–2010 using this method is 0.77 Pg C annum^−1^. If we use instead the recent and probably more reliable satellite-based deforestation figures (Hansen *et al.*, 2013) instead of the FAO figures, we obtain a higher value of 0.93 Pg C annum^−1^ (Table 5). The regional totals from FAO and Hansen are far from being in agreement, except for South America where the data are dominated by Brazil, a country where the deforestation rate reported to the FAO is estimated by remote sensing and where the agreement between the FAO data and Hansen's data is much better. On the other hand, the deforestation rates of most African countries are less than the FAO figures, often by a margin exceeding 50%, while in Asia the discrepancy is the other way around (the Hansen deforestation rates of Indonesia and Malaysia are more than double the reported rates).

**Table 5:** Estimated annual carbon flux from tropical deforestation (FAO, 2011). Comparison is made with the FAO figures using carbon density data from Saatchi to convert areas to carbon stocks (FAO-S), and satellite data from Hansen *et al*. (2013) over a similar period (2000–2012) also using carbon density data from Saatchi *et al*. (2011) to convert areas of forest loss into carbon fluxes. The final column is an estimate of the degradation flux (see text). Units PgC annum^−1^

	FAO 1990–2000	FAO, 2000–2005	FAO 2005–2010	FAO-S 2000–2010	Hansen 2000–2012	Degradation flux 2000–2012
America	0.45	0.45	0.38	0.46	0.48	0.05–0.24
Africa	0.28	0.27	0.27	0.21	0.12	0.01–0.06
Asia	0.35	0.05	0.14	0.098	0.33	0.03–0.15
Total	1.09	0.79	0.80	0.77	0.93	0.09–0.46

In constructing the trends over time in the carbon budget in any scenario of deforestation, it must be kept in mind that the carbon in the trees and litter is not all immediately oxidized. Some trees may survive and die later; and below-ground components, once dead, decompose rather slowly at a rate which is likely to vary enormously with the wood composition, the moisture content of the soil and the fineness of the dead material. In the analysis given above, we have made no attempt to model the decomposition rate, although some authors have done so (Gloor *et al.*, 2012; Houghton *et al.*, 2012).

##### *F*_Degradation_

‘Forest degradation’ refers to a loss of biomass which is not visible by conventional remote sensing, and which usually goes unreported. It arises mostly from selective logging, where the fraction of trees removed is not sufficient to change the land cover from ‘forest’ to ‘non-forest’ (Nepstad *et al*. 1999; Asner *et al.*, 2005). It may also occur as a result of fire or drought, where damage occurs in the subcanopy and the large trees are relatively undamaged and so the canopy viewed from space is identified as ‘forest’. It may also be associated with fragmentation, the process whereby the forest is broken up into small subunits which may then be exposed to ‘edge effect’ and become more susceptible to drought. Measuring degradation has been attempted for specific regions. For example, in the Congo Basin. Ernst *et al*. (2013) found that the forest area affected by degradation was of a similar size as the deforested area and commented that deforestation and degradation were usually interrelated. At present, insufficient information is available to estimate the tropical degradation flux and it may be the largest uncertainty in the tropical carbon budget. Here, we assume that the degradation flux is between 10% and 50% of the deforestation flux, yielding an estimate of 0.27 ± 0.11 Pg annum^−1^. In the future, it is expected that radar remote sensing will provide regular information on biomass as well as forest cover, and so the uncertainty in degradation flux may be reduced (Le Toan *et al.*, 2011).

**Figure 3:**
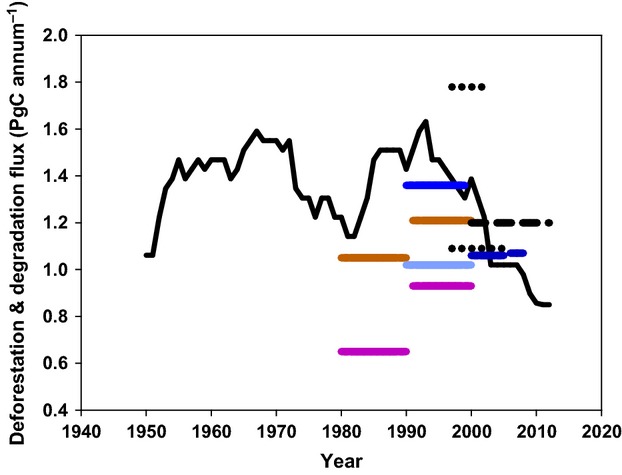
Estimates of the carbon flux from deforestation and degradation in the tropics. The solid black line is redrawn from Houghton *et al*. (2012). From the current analysis: black dashed line from the data of Hansen *et al.*, 2013; the dark blue lines from calculations using the FAO data. The remaining lines are redrawn from the synthesis by Van der Werf *et al*. (2009) as follows: purple, DeFries *et al.*, 2002; black dots, IPCC working group III (Barker *et al.*, 2007; Nabuurs *et al.*, 2007); pale blue, Achard *et al.*, 2002; brown, IPCC working group I (Denman & Brasseur, 2007).

Using Hansen's data, we estimate deforestation plus degradation fluxes of 1.20 ± 0.17 Pg annum^−1^, yielding total fluxes which are more or less consistent with the many data sets reviewed by Houghton *et al*. (2012) and the estimate of ‘about 1.2’ Pg C annum^−1^ by Van der Werf *et al*. (2009) for the emissions associated with ‘deforestation and degradation’. In Fig.3, we combine the data of Van der Werf *et al*. (2009), Houghton *et al*. (2012) and the estimates obtained in this study as *F*_Deforestation_ + *F*_Degradation_ using either the FAO data or the Hansen *et al*. (2013) data. We see large discrepancies, but the most recent results (which may be more reliable) provide support for van der Werf's estimate of 1.2 Pg C annum^−1^.

#### *F*_Plantation_, the growth of plantation flux

A proportion of the forest detected in each region is in the form of plantations, which vary enormously in their growth rates (Nambiar, 2008). Some of these are in a stage of particularly rapid growth: Laclau *et al*. (2000) found growth rates of 16 Mg biomass ha^−1^ annum^−1^ for eucalyptus in the Congo, and much higher rates are possible with appropriate silviculture (Stape *et al.*, 2010). But other tree species are slow growing, including those producing high-value timber such as teak and mahogany. One of the fast-increasing types of plantation is palm oil, motivated by the goal to use the oil as fuel, thus to reduce fossil fuel emissions (Gibbs *et al.*, 2008 Achten & Verchot, 2011; Obidzinski *et al.*, 2012). Rubber plantations have also increased; they have expanded rapidly in tropical parts of China (Yunnan and the island of Hainen). Like palm oil, there are concerns about the destruction of species-rich forests in these parts, and the possible degradation of soil (Cheng *et al.*, 2007; Li *et al.*, 2007, 2008; Zhai *et al.*, 2012; Song *et al.*, 2013).

Food and Agriculture Organization figures (FAO 2010) suggest that plantations in the tropics have increased from 90 million ha in 1990 to 144 million ha by 2010. To estimate the impact, they are having on the tropical carbon budget we have run a simple model in which the biomass of plantation forests increases sigmoidally to reach 120 MgC ha^−1^ after 100 years (consistent with Ziegler *et al.*, 2012); we further assume a constant planting rate of 2.44 million ha per year from 1960 to 2010 (matching FAO figures), and then we track the annual cohort until 2010, finally adding all the cohorts. In this scenario, plantations develop sink strength of 0.24 PgC annum^−1^ by 2010 and accumulate a stock of 5.48 PgC. However, in achieving this state, the native forest they replace has been lost: we estimate the loss has been 15 Pg of carbon altogether. If we take a much less favourable scenario, with lower growth rates and shorter stand cycles, the sink is lower, as little as 0.10 PgC annum^−1.^ These figures could be refined if reliable information from country-level inventories and management regimes were to be made available.

#### *F*_Secondary_, the flux from the regrowth of secondary forest

A secondary forest is a forest or woodland which has regrown after a major disturbance (fire, wind-throw are natural disturbances, but the use of the land for agriculture is the major anthropogenic disturbance in the context of this review, see Chokkalingam & de Yong, 2001). In the tropics, the regrowth usually reaches the biomass of the original forest in 100 years. The biodiversity recovers more slowly (Martin *et al.*, 2013), but can be remarkably high (Berry *et al.*, 2010).

Much of all tropical forest is now secondary forest (*sensu* Brown & Lugo, 1990). According to FAO (2010) about 88% of Africa's tropical forest is now secondary; the corresponding figures for Asia and South America are 62% and 23%. This includes forest developing on abandoned farmland, forest regrowing from having been destroyed or logged and woody encroachment into savanna (Mitchard & Flintrop, 2013). Secondary forest often accumulates carbon rather rapidly when young and then more slowly (Brown & Lugo, 1990; Sierra *et al.*, 2012). In a recent analysis of data from all three tropical continents, Bonner *et al*. (2013) found carbon uptake rates of secondary forest from 0.25 to 6 MgC ha^−1^ annum^−1^, with a central tendency of about 2.5 MgC ha^−1^ annum^−1^, not very different from the value obtained from the much earlier (but smaller) data set by Brown & Lugo (1990). Working in forests on poor soils at Chiapas, Mexico, Orihuela-Blemonte *et al.,* (2013) found lower rates: the average uptake rate of carbon over 40 years, including both live and dead organic matter and also soil carbon, was 2.66 MgC ha^−1^ annum^−1^, and the rate declined over three successive cycles of slash and burn.

Using the FAO figures on the extent of secondary forest, we may estimate the uptake of carbon from these forests as being from 0.8 PgC to 1.6 PgC annum^−1^. This value is consistent with the tropical regrowth sink of 1.6 ± 0.5 PgC annum^−1^ proposed by Pan *et al*. (2011). In the past, many authors have neglected the secondary forest sink and focussed attention on the sink in the primary forests (Lewis *et al.*, 2009; Wright, 2013), despite the availability of remotely sensed and inventory data on the extent of secondary forests, and its increasing importance as the expanding agricultural land reaches the point of abandonment (Lucas *et al.*, 2000; FAO, 2010; Aragão *et al*., 2014).

Of all the tropical continents, Africa is the most difficult to analyse in terms of the areas of secondary vs. primary forest because its land cover and land use is the most complex, and the science infrastructure is considerably weaker than elsewhere. Recent reviews (Bombelli *et al.*, 2009; Ciais *et al.*, 2010; Valentini *et al.*, 2013) have brought the area into sharp focus and it is hoped that the research effort in Africa will eventually match that in Latin America. Presently, Africa remains one of the largest sources of uncertainty in our attempt to produce a tropical carbon budget. The most recent analysis concludes that land-use emissions for Africa amount to 0.32 ± 0.05 Pg C annum^−1^, while the African continent as a whole is a small but uncertain net sink because the accumulation of carbon in forests and woodlands exceeds the land-use emissions (Ciais *et al.*, 2010; Valentini *et al.*, 2013).

#### *F*_Primary_, sink in the intact forest

The adjectives ‘intact’, ‘pristine’ and ‘virgin’ are used almost interchangeably to describe forest which has not been disturbed in living memory. Here, we use ‘intact’ acknowledging that ‘intact’ forest is neither pristine or virgin, having been disturbed in various ways by humans over thousands of years (including subsistence agriculture based on slash and burn which may have occurred hundreds of years ago, see Whitney *et al.*, 2014).

It was formerly considered that intact forest is more or less at a steady state (e.g. Odum, 1966). However, a global data set suggests it is close to but not exactly in steady state, and that even old forests accumulate carbon at a measurable rate (Luyssaert *et al.*, 2008). This may be the result of a CO_2_ fertilization effect, for reasons enunciated by Lloyd & Farquhar (1996) and others, or there may be other reasons or artefacts pertaining to how forest plots have been selected and sampled (Fisher *et al.*, 2008). The data compiled by Lewis *et al*. (2009) from primary forests on three continents suggest a very large pan-tropical forest sink of 1.3 Pg C annum^−1^, which these authors tentatively attributed to CO_2_ fertilization. In making their estimate, Lewis *et al*. (2009) estimated a rate of carbon accumulation of 0.49 MgC ha^−1^ annum^−1^ from a large network of forest plots which were ‘undisturbed’. If we apply that rate over the pan-tropical areas which are intact forests (a smaller area than that used by Lewis *et al.*, 2009 who seems to have lumped some secondary forests with primary forests), we obtain an estimate of the pan-tropical sink strength in primary forests of 0.47 Pg annum^−1^.

#### *F*_Harvest_, the harvested products

Tropical forests are harvested (i) to provide wood-fuel and charcoal at the local level and (ii) for timber and other wood products that may be exported. Wood for fuel constitutes 70% of the total wood harvest (FAO, 2011). The two categories have different average lifetimes, but here we assume that both are destined to be converted to CO_2_ immediately. In harvesting timber, the species that are highly valued for their strength, appearance and durability are selected. When harvesting wood-fuel there is still some selection but it is less. The gathering of wood for fuel, or for conversion into charcoal to sell in markets, is traditionally less than the biological wood production, but in sparse African woodlands this is not always the case and villagers walk far to seek fuel-wood, consuming about 1 m^3^ of wood per person per year (Zimmerman & Kormos, 2012).

To calculate the carbon flux from harvesting, we assume that one cubic metre of timber contains 0.25 tonnes of carbon. The tropical timber harvest flux calculated from this method, derived from the harvested volumes published in FAO (2011) is 0.34 PgC annum^−1^, most of it being fuel-wood. If allowance is made for wastage (assume a maximum of 50%) at the saw mill, this figure increases only slightly to 0.36 because only a small fraction of the harvested product is destined for the saw mill. Some of the harvested timber is for export markets and so part of the emissions from this source may occur outside the tropics.

#### *F*_Peat_, the flux from the loss of tropical peat lands

Large quantities of peat have been deposited in some areas of the humid tropics over thousands of years (Page *et al.*, 2011). The tropical peat-land area is thought to be over 441 000 km^2^ (i.e. 11% of the global peat-land area) of which more than half is in south-east Asia. In the undisturbed state, the peat deposits are assumed to decompose very slowly, so slowly that over a year their contribution to emission can be assumed to be zero. But in the process of conversion from undisturbed rain forest to industrial plantations, this peat is drained and exposed to aerobic conditions; in some cases it may burn and smoulder for years. It thus produces substantial emissions of CO_2_ directly to the atmosphere and may also lose carbon in the drainage waters (Moore *et al.*, 2013). The extent of this loss has been estimated as high as 20 MgC ha^−1^ annum^−1^ from measurements of subsidence at specific research sites (Hooijer *et al.*, 2010, 2012). Of the 27.1 Mha of peat land in south-east Asia, Hooijer *et al*. (2010) stated that 12.9 Mha had been deforested and mostly drained by 2006. Thus, we may expect this deforestation would lead to an average efflux of some 0.54 PgC annum^−1^, a large figure which has been overlooked by most researchers. Very large losses may occur in some particular years: Page *et al*. (2002) estimated that between 0.81 and 2.57 Gt of carbon were lost in 1997 as a result of peat fires in Indonesia.

#### *F*_Fire_, the fire flux

Large amounts of CO_2_, CO and CH_4_ and black carbon are released into the atmosphere during biomass burning (Seiler & Crutzen, 1980; Van der Werf *et al.*, 2003; Scholes *et al.*, 2011). However, most of the carbon fluxes have already been accounted for above in our analysis of *F*_d_, the deforestation flux.

There are also fluxes from shifting agriculture in regions where the natural vegetation can be either secondary forest or savanna. These result from the clearing of the woody vegetation which has developed over periods that may vary from 2 to 30 years. In a most detailed analysis, Silva *et al*. (2011) examined the fluxes of greenhouse gases from shifting agriculture in the tropics. Using FAO agricultural statistics and land areas from the Global Land Cover 2000 (GLC2000) data set, they estimate that carbon fluxes from the tropics were as high as 0.20 PgC annum^−1^ though any losses from burning might be expected to be offset by the carbon sequestration in recovering fallows, as Silva *et al*. (2011) acknowledged, but only in the final sentence of their article.

In burning, not all of the biomass carbon is converted to gaseous form. Working in the Amazon forest, Fearnside *et al*. (2001, 2007) found that 1.8% of the above-ground carbon remained as charcoal. Its fate is not well-known but some information may be gleaned from consideration of Brazil's Atlantic forest. This forest was largely removed between the 1850s and the 1970s, but the black carbon from charcoal stored in the soil continues to be found today in the drainage water (Dittmer *et al.*, 2012).

During burning, carbon particles enter the atmosphere as smoke, and are widely dispersed over vast regions of land and ocean. They are resistant to biological decomposition, but probably not as resistant as was thought previously (see Bird *et al.*, 1999). This carbon flux was estimated by Kuhlbusch & Crutzen (1996) to be 0.05–0.20 PgC annum^−1^, but a recent estimate (which we adopt in this analysis) suggests a much lower figure of 0.007 PgC annum^−1^ (Bond *et al.*, 2013). It should nevertheless be kept in mind that elemental carbon, whether as charcoal or smoke, is sometimes considered to be a carbon sink as it represents transfer from the relatively volatile form (biomass) to a relatively stable form (elemental carbon). On the other hand, the presence of black carbon in the atmosphere is probably a major contributor to global warming (Bond *et al.*, 2013).

### Sum of the CO_2_ fluxes

**Figure 4:**
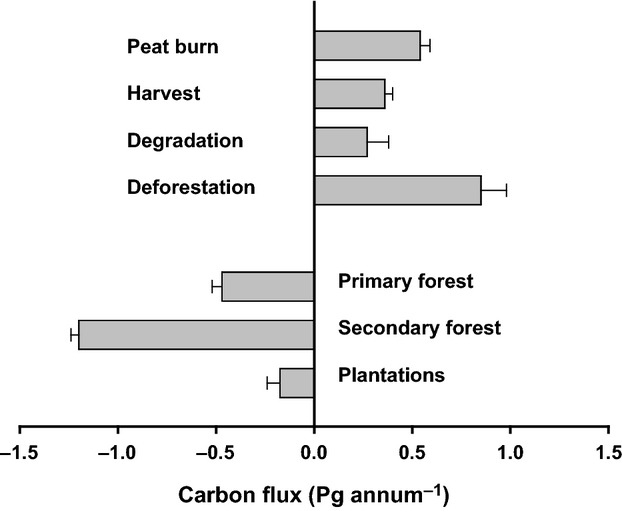
Components of human-induced change in the tropical biological carbon balance, with uncertainties. Negative denotes uptake from the atmosphere to the land surface, positive denotes loss of carbon to the atmosphere. The data are applicable to the period 2005–2010.

The sum of the sink terms *F*_Plantation_, *F*_Secondary_, *F*_Primary_ is 1.85 ± 0.09 Pg C annum^−1^, while the sum of the source terms *F*_Deforestation_, F_Degradation_, *F*_Harvest_ and *F*_Peat_ is 2.01 ± 1.10 Pg annum^−1^, making the tropics a net carbon source of 0.16 Pg annum^−1^ with an uncertainty of about ± 1.1 (Fig.4). Considering the uncertainties, we may conclude that the land surface is nearly carbon neutral, but could be a strong sink if deforestation and degradation were to cease.

It may be useful to compare these data with the emissions from fossil fuel burning. For tropical countries, this now amounts to 0.74 PgC annum^−1^ (Boden *et al.*, 2010). The *per capita* fossil fuel emissions of tropical countries are closely related to economic activity and trade (DeFries *et al.*, 2010) and have increased sharply since 2004 suggesting that those tropical countries that have achieved significant economic development have done so by burning fossil fuels rather than by using biomass. The average annual *per capita* emissions of the tropical countries in this study is 0.57 MgC person^−1^, much lower than the corresponding figures from developed regions of the world (Australia is 5.0, USA is 4.6, UK is 2.09 MgC person^−1^ annum^−1^), and lower than the global average of 1.30 MgC person^−1^ annum^−1^.

## The atmospheric signal

The carbon cycle is ‘boundless’ as several authors have emphasized (Battin *et al.*, 2009) and site-related (ecosystem) studies suffer from the fact that sampling field plots across the entire world in a proper statistical manner is an impossibility. Not only is vegetation enormously diverse in its natural formations and even more so in its disturbed state, but also there are lateral flows of dissolved carbon that we are only beginning to understand (Richey *et al.*, 2002; Grace & Malhi, 2002). At best, plot-based studies such as those discussed above may be used to reveal component processes and define underlying trends which can be associated with climatological variation, and used to calibrate models (Friend *et al.*, 2007).

An alternative approach is to estimate sources and sinks from the effect the land surface has on the concentration of CO_2_ in the atmosphere. This can be done by using (i) measurement of CO_2_ concentration from a global network of points followed by an approach known as ‘atmospheric inversion’, a procedure first realized by Bolin & Keeling (1963), then developed by Enting & Mansbridge (1989) and Tans *et al*. (1989) or (ii) examination of many profiles of CO_2_ in the planetary boundary layer, achieved by aircraft flights (Chou *et al.*, 2002; Gatti *et al.*, 2010). Interpretation of the data is greatly enhanced by the use of isotopic concentrations: fluxes over land and ocean can be distinguished by their carbon isotopic signal *δ*^13^C, as photosynthesis favours uptake of ^12^CO_2_ against ^13^CO_2_, while the purely physical gas exchange between the atmosphere and the ocean is isotope-indiscriminate. Thus, the contributions to global sources and sinks made by ocean vs. the land may be compared.

Initially, research groups working at global scale developed their own computational procedures to estimate fluxes from concentrations, but in the late 1990s there were strong attempts to work together (Gurney *et al.,* 2004). Data on the global distribution of fossil fuel emissions (Boden *et al.*, 2010) are subtracted from the calculated terrestrial fluxes to reveal an estimate of the natural carbon fluxes associated with photosynthesis, respiration and air-sea exchange. Gurney *et al.,* (2004) and Peylin *et al*. (2013) have reported the results from several different models covering the periods 1992–1996 and 2002–2008 respectively (Table 6). Both results say that the northern hemisphere contains a large land sink of carbon, of order 2.0 Pg annum^−1^, usually attributed to the extensive areas of growing forests and plantations in the temperate and boreal regions. The uncertainty term is much lower than the estimate. But for the tropics, the uncertainty is generally similar to, or greater than, the estimate. For example, of the 11 models reported by Peylin *et al*. (2013) one indicates the tropics to be a land sink, four suggest the tropics to be a very weak source, and five show a source greater than 1 Pg annum^−1^. The main reason for the large uncertainty from the inversion result is the small number of sampling stations located in the tropics. Another possibly reason is that the biological components of carbon flux are highly sensitive to temperature and drought, and thus vary across sampling periods and across sampling space.

**Table 6:** Global sources and sinks according to atmospheric inversions, not counting the fossil fuel emissions. The stated values are the mean of several models followed by ± Standard Deviation (often used as the measure of uncertainty). Units: PgC annum^−1^

Authors	Period	Global		North		Tropical		South	
		Land	Ocean	Land	Ocean	Land	Ocean	Land	Ocean
Gurney *et al.,* (2004)	1992–1996	−1.4 ± 0.8	−1.5 ± 0.8	−2.3 ± 0.8	−1.3 ± 0.6	1.1 ± 1.1	0.3 ± 0.5	−0.2 ± 0.7	−0.8 ± 0.5
Peylin *et al*. (2013)	2001–2004	−1.5 ± 0.6	−1.6 ± 0.5	−2.2 ± 0.5	−1.1 ± 0.3	0.9 ± 1.0	0.8 ± 0.2	−0.1 ± 0.4	−1.3 ± 0.3

Several groups have inferred fluxes from vertical profiles of concentration using aircraft (Chou *et al.*, 2002; Lloyd *et al.*, 2007; Gatti *et al.*, 2010, 2014). Many such data were compiled by Stephens *et al*. (2007), who found evidence for a rather different balance between tropical and northern sinks: they suggested that the temperate zone is less of a sink than previously thought, while the tropics may be a significant sink.

A recent aircraft study provides insights into the behaviour of the carbon sink in the Brazilian Amazon (Gatti *et al.*, 2014). Vertical profiles of CO_2_ up to 4 km showed marked differences between the burning season (July–October) when the surface concentrations were enriched by several parts per million of CO_2_. In the rest of the year, photosynthesis by the vegetation drew down the surface concentration below the background. By contrasting the year 2010 (a drought year) with 2011 (a more normal year), and by also measuring carbon monoxide (a marker for fire), the researchers were able to separate the impact of drought on the basic biological process and on the fire occurrence. In the drought year, the uptake of carbon dioxide by the vegetation was reduced by 0.22 PgC. Overall, the Amazon basin was changed from being more or less carbon neutral in the normal year of 2011 to being a source of 0.48 PgC of carbon in the drought year.

## Sensitivity of the flux to climate change

The question of whether the carbon balance of the land surface changes from year to year, and whether there are long-term changes associated with global warming remains highly controversial since the modelling paper by Cox *et al*. (2000), which predicted that the rainforest would be converted to savanna as a result of warming, with the loss of substantial stocks of soil carbon, a process involving a positive feedback on global warming caused by the additional release of CO_2_. From a physiological viewpoint, the evidence at that time was slim, and even now there are still only a few long-term experiments that have a bearing on the issue (Wood *et al.*, 2012). Reliance on models which make simplified assumptions without any experimental evidence seems ill-advised, as matters such as the effect of elevated CO_2_ on photosynthesis and the effect of warming on soil respiration in the long term are not properly established. Indeed, a recent report of model outputs tends to rebut Cox's notion: only one of 22 models suggests that tropical rain forests will decline by the end of the century (Huntingford *et al.*, 2013). There are however a few lines of enquiry which do not rely on models, but on empirical evidence, as outlined below.

Eddy covariance data of CO_2_ fluxes over tropical ecosystems may be used in an attempt to investigate the sensitivity of those fluxes to changes that occur naturally as a result of changing weather patterns or seasons. Using a statistical model fitted to a very limited data set, Grace *et al*. (1995) found that small increases in temperature turned the forest from a modest sink of carbon to a large source, as a result of the effect of temperature on respiration, especially soil respiration. Most of studies on gas exchange of vegetation and soils have been short term, particularly those on soil respiration. For soils, longer term and larger scale observations suggest a somewhat different outcome from that obtained in short campaigns, because over a period of weeks and years the microbial populations change in a process which is (speculatively) termed ‘acclimation’ (Giardina & Ryan, 2000; Grace & Rayment, 2000; Knorr *et al.*, 2005). This may involve shifts in the quantity and quality of the organic matter available for the microbial population (Davidson & Janssens, 2006). Perhaps new microbes with different temperature sensitivities establish a strong presence in the forest soil.

The respiratory fluxes from soil are large (Fig.2). One review of the available tropical data reported annual rates of soil respiration of 3–6 μmol CO_2_ m^−2^ s^−1^, equating to 11–22 MgC ha^−1^ annum^−1^ compared to typical intact forest growth rates of 2–4 t ha^−1^ annum^−1^ (Sotta *et al.*, 2004). Some of this flux is autotrophic respiration, originating from plant roots, but about half is heterotrophic respiration arising from the microbial breakdown of organic matter (Butler *et al.*, 2012). This breakdown releases nitrogen and phosphorus from organic compounds and so is important not only because of CO_2_ release but also because of the possible stimulatory role in plant nutrition. In short-term experiments (hours, days) the rate of respiration is a more or less exponential function of temperature over the normal environmental range (Lloyd & Taylor, 1994). Given that the observed temperature in the tropics has increased by about 0.5 °C since 1950, and is set to increase even more (IPCC 2007), it seems likely that carbon efflux from the soil has been increasing over the last 50 years and will continue to do so. Wood *et al*. (2012) draw no firm conclusions about the effect of temperature on the soil carbon efflux but instead they call for long-term field experimentation in the tropics.

Some longer term manipulation experiments do exist. There have been two long-term (at least 7 years) drought experiments in the Amazon where about half the annual rainfall was excluded by means of shelters. Nepstad *et al*. (2007) reported an increased mortality of trees. In a very similar but independent experiment, Da Costa *et al*. (2010) demonstrated that 38 MgC ha^−1^ were lost over seven years (i.e. 5.4 MgC ha^−1^ annum^−1^) and an increased mortality of trees occurred. Individual years of extreme drought, like 2005 and 2010, are likely to cause large carbon losses according to estimates by Phillips *et al*. (2010) quite apart from any direct adverse effects of associated high temperatures.

There are rather few recent attempts to measure directly the effect of changing temperature on the growth of tropical trees (Clark *et al.*, 2013; Vlam *et al.*, 2014). They show a decline in growth rate with increasing temperature.

Modelling the effect of rising temperature on the balance between photosynthetic uptake and respiratory losses by ecosystems is challenging because there are many processes to be considered as components of ‘ecosystem metabolism’ (Malhi, 2012): the photosynthetic uptake is likely to interact with water, nutrient and CO_2_ supply, while the respiratory losses involve both autotrophic and heterotrophic components which are likely to be especially sensitive to water and nutrient supply, but not in the same way as photosynthesis (Lloyd & Farquhar, 1996). The temperature effect on soil respiration over long periods appears to be quite different from the exponential relation found in short-term experiments (Giardina & Ryan, 2000; Grace & Rayment, 2000; Knorr *et al.*, 2005). Thus, modelling is not sufficiently reliable to establish a firm link between temperature and ecosystem carbon storage.

One approach towards exploring the temperature sensitivity of the carbon cycle over the long term is to investigate carbon stocks along well-defined geographical gradients. In a meta-analysis of pan-tropical data from three continents, Raich *et al*. (2006) demonstrated that soil organic matter decreased by 8 MgC ha^−1^ for every degree Kelvin, while plant biomass increased by 5.2 MgC ha^−1^ K^−1^. The differences between these two fluxes, 2.8 MgC ha^−1^ K^−1^, may be speculatively considered to be a measure of the extent to which carbon accumulation falls with temperature in the tropics. Up-scaling this temperature coefficient to the entire area of tropical forests (including primary, secondary and plantations, about 20 million km^2^) suggests a carbon loss of 5.6 Pg for every degree of warming. Such a large signal should be evident in the atmospheric data. To some extent it is: Langenfelds *et al*. (2002) showed that the interannual variations in global atmospheric CO_2_ concentrations were associated fluctuations in *δ*^13^CO_2_, showing the importance of terrestrial vegetation in the carbon cycle. They further demonstrated that years with high CO_2_ were associated with high CH_4_, CO and H_2_, all gases coming from biomass burning. Later, Heimann & Reichstein (2008) showed that such high CO_2_ years were associated with large scale *El Niňo* influences. The causal processes in that case may be: *El Niňo* → Drought and high surface temperatures → Fire → Carbon Loss. This chain of cause-and-effect may be more important than: Warm year → High respiration and Low photosynthesis → Carbon Loss.

Models, however preliminary, provide a means to begin the integration of knowledge and to upscale the information to reveal the bigger picture. Cox *et al*. (2013) present the most recent attempt to use climate-carbon models to infer the sensitivity of the tropical carbon cycle to warming. Seven such models were run, and their results showed considerable scatter; however the authors were able to conclude that warming is likely to release 53 ± 17 Pg carbon per degree Kelvin over the period 1960–2099. Assuming two degrees of global warming, the annual increase in emissions would therefore be substantial, at 0.76 PgC annum^−1^.

## Concluding remarks

The perturbations to the tropical carbon cycle brought about directly by human activities cause about two million tonnes of carbon per year to be added to the atmosphere as CO_2_. We have shown how this loss of carbon is more or less balanced by the strong forest sink. Based on our analysis, it is difficult to deny Pan's assertion of a ‘large and persistent carbon sink in the world's forests’ (Pan *et al.*, 2011), although Wright has recently tried to do so (Wright, 2013). There seems little doubt that the combination of primary and secondary forest produces a sink approaching 2 PgC annum^−1^ in the tropics. The contribution of the secondary forests has not been fully recognized previously, and seems to have been overlooked (Wright, 2013).

To what extent is the knowledge and understanding of the tropical carbon budget now adequate as the basis for REDD and REDD+ projects? The important technical advances made recently have been in satellite remote sensing, which has delivered moderately high resolution data on forest cover change (Hansen *et al.*, 2013) and promises to provide data on biomass from radar backscatter (Le Toan *et al.*, 2011). Previously, the technical challenge of doing this and the inadequacy of inventory-based reporting were often cited as an obstacle to the progress of REDD+. Future developments in remote sensing, outlined above, promise additional capability to detect change at scales that are appropriate to assessment of change in the global carbon cycle and even to monitor quite small, community-based, carbon projects (Mitchard *et al.*, 2013a,b). It remains important to make such data easily available and free to governments and land managers.
